# Development and validation of prognostic prediction model for submandibular gland cancer based on the SEER database

**DOI:** 10.1016/j.bjorl.2025.101654

**Published:** 2025-06-25

**Authors:** Junkun He, Feng Zhao, Jiangmiao Li, Qiyun Li, Fangyu Wei, Jiping Su

**Affiliations:** Department of Otolaryngology, Head and Neck Surgery, The First Affiliated Hospital of Guangxi Medical University, Nanning, China

**Keywords:** Submandibular gland carcinoma, SEER, LASSO regression, Cox proportional hazards model, Nomogram

## Abstract

**Objective:**

Accurately predicting the prognosis of Submandibular Gland Carcinoma (SGC) patients remains a challenging task. The purpose of this study was to develop a columnar graph prognostic prediction model for submandibular gland cancer based on the SEER database, using feature selection with lasso regression and modeling with Cox regression.

**Methods:**

This study utilized data from the SEER database, focusing on 1362 cases of SGC. Various clinical and demographic factors, including age, tumor size, histology, and lymph node metastasis, were considered as potential prognostic factors. Feature selection was performed using lasso regression, and a Cox proportional hazards model was constructed, taking into account the complex interactions between variables and their impact on survival outcomes.

**Results:**

The established prognostic prediction model demonstrated good accuracy and reliability. The model effectively identified several important prognostic factors, including age, tumor size, histology, and lymph node metastasis, which strongly influenced the prognosis of SGC. The model showed good discrimination and calibration with c-indexes of 0.802 (0.784‒0.821) in the training set and 0.756 (0.725‒0.787) in the validation set. The Decision Curve Analysis (DCA) curve reflected clinical utility.

**Conclusion:**

This study suggests that the prognostic prediction model based on Cox regression is a valuable tool for predicting the prognosis of patients with SGC. This approach has the potential to improve patient outcomes by facilitating personalized treatment plans and identifying high-risk patients who may benefit from more aggressive interventions.

**Level of Evidence:**

Level III.

## Highlights

• Prognosis of submandibular gland carcinoma remains challenging to predict.
• Lasso regression selected key prognostic factors.
• Cox regression model established for prognostic prediction.
• The model demonstrated accuracy with c-indexes in training and validation sets.
• Decision curve analysis confirmed the model’s clinical utility.

## Introduction

Submandibular Gland Carcinoma (SGC) is a rare but impactful type of malignancy within the head and neck region. Salivary gland cancers account for approximately 6.5% of head and neck cancer cases,^1^ with SGC comprising 7%–11% of salivary gland tumors, of which 30%–54% are malignant.^1^, ^2^ Despite its relatively low incidence, the impact of submandibular gland cancer on patient survival and quality of life cannot be overlooked. SGC cancers exhibit various histological subtypes, including Mucous Epidermoid Carcinoma (MC), Adenoid Cystic Carcinoma (ACC), and Adenocarcinoma (AC), among others.^3^ Due to their anatomical location, potential for invading neighboring structures, and tendency for local recurrence and distant metastasis, they may present diagnostic and treatment challenges.^4^

Epidemiological studies have revealed the characteristics of SGC. Although these tumors constitute a small proportion of salivary gland tumors, their specific epidemiological features provide valuable insights. The primary treatment for malignant salivary gland tumors remains surgery, although adjuvant radiotherapy may play a significant role in patients with advanced disease.^5^, ^6^ Neutron, heavy ion, or proton radiation therapy may be a treatment option for locally unresectable disease. Depending on the location of metastatic lesions, metastatic disease can be treated with radiation therapy or palliative chemotherapy.^7^ A study based on the Surveillance, Epidemiology, and End Results (SEER) database reported that age, tumor grade, TNM stage, gender, surgical size, tumor size, and radiotherapy were associated with survival rates.^6^

In recent years, there have been some advancements in the development of prognostic prediction models for salivary gland cancer, aiming to provide accurate estimates of survival outcomes and facilitate personalized treatment decisions. For instance, Ran et al. and Luo et al. have separately constructed competing risk models for salivary duct carcinoma and parotid carcinoma based on the SEER database.^8^, ^9^ However, there is currently no specific prognostic prediction model developed specifically for SGC.

The aim of this study is to develop and validate a prognostic prediction model for SGC based on the Surveillance, Epidemiology, and End Results (SEER) database. The SEER database is a valuable resource of cancer epidemiological data in the United States, containing clinical characteristics, treatment information, and survival outcomes of a large number of submandibular gland cancer patients. By utilizing this valuable database, we will construct a predictive model that comprehensively considers clinical features and prognostic indicators to accurately predict Overall Survival (OS) in SGC patients.

In the model construction process, we will employ a combination of LASSO (Least Absolute Shrinkage and Selection Operator) and Cox proportional hazards models. Additionally, columnar graphs have been widely used in various cancer prognosis studies, integrating the effects of multiple predictive factors into a simple scoring chart for convenient assessment of individualized prognosis by clinical practitioners.

Through the development and validation of this prognostic prediction model for SGC based on the SEER database, we aim to provide clinicians with accurate prognostic assessment tools and personalized treatment recommendations to improve the survival rate and quality of life of SGC patients. Furthermore, we will comprehensively evaluate and validate the model's performance to confirm its feasibility and accuracy in real-world clinical applications.

## Methods

### Study population

The data were extracted from the SEER Researcher Plus Database (Nov 2020 Sub). All data supporting the findings of this study are available within the paper and its Supplementary Information. Supplementary Table 1 provides all the raw data downloaded from the SEER database. Inclusion criteria were as follows: 1) Site and morphology codes were classified as “submandibular gland cancer”; 2) Analyzed behavior records were malignant. Exclusion criteria were: 1) Lack of cause of death information; 2) Absence of follow-up survival months or a survival time of 0-months; 3) Patients with insufficient clinical information, such as surgery details, primary site codes, AJCC stage (AJCC 7th edition), or TNM stage (AJCC 7th edition). The endpoint of this study was Overall Survival (OS), defined as the time interval from the initial diagnosis to death.

### Variables

**Fig. 1:**
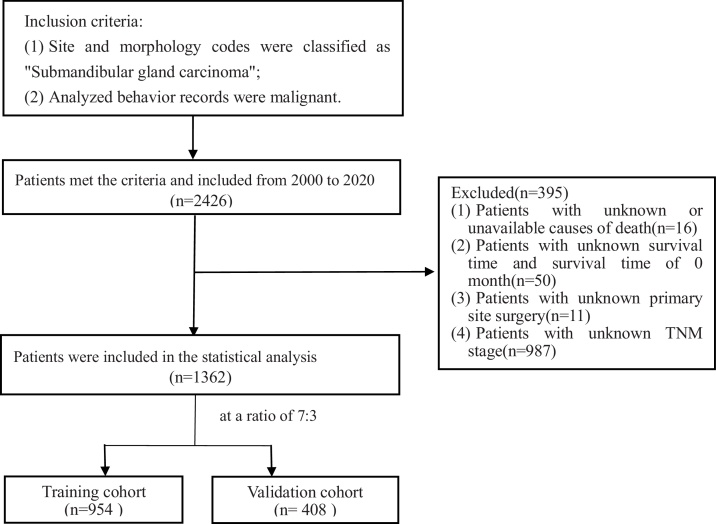
The process of research design.

The following variables were collected: demographic information, including age, race, gender, and marital status; inclusion of clinical characteristics, including laterality, grade, AJCC stage (AJCC 7th edition), T-stage, N-stage, M-stage, tumor size, and histological type. Additionally, this study incorporated records of surgery, radiotherapy, chemotherapy, and others. Using the X-tile software for analysis, we set the optimal age cutoff points for overall survival time at 60-years and 80-years, categorizing patients into three age groups (<60, 60–79, and ≥80-years). The selection details of the study subjects are illustrated in the flowchart (Fig. 1).

### Model establishment

Patients were randomly divided into a training set and a validation set in a 7:3 ratio, and variable selection was performed before modeling. The model employed Least Absolute Shrinkage and Selection Operator (LASSO) regression analysis to select variables in the training set. If a variable showed statistical significance (*p* < 0.05), it was included in the multifactor Cox regression for further screening. Finally, a Cox proportional hazards model was constructed, and a nomogram was established based on this model.

### Model evaluation and interpretation

The performance of the model was evaluated in both the training and validation sets. The predictive accuracy was assessed using the Concordance Index (C-index) and the Area Under the operating Characteristic Curve (AUC) at 1-, 3-, and 5-years.^10^ The calibration ability was evaluated using calibration curves, and a well-calibrated model would show points close to the 45 ° diagonal line on the calibration plot. Clinical net benefit of the model was assessed using Decision Curve Analysis (DCA).

### Patient risk stratification

Patient risk stratification is based on risk scores, calculated through nomogram, to quantify the survival risk of patients. Using *R* software, we classified patients into high, medium, and low risk groups based on their risk scores. Kaplan-Meier survival analysis and log-rank tests were employed to assess the risk stratification.

### Statistical analysis

Statistical analysis was conducted using *R* software version 4.3.1 (http://www.R-project.org). The following packages were utilized: CBCgrps, glmnet, survival, rms, mlr3, and rsf, among others. A two-tailed *p*-value <0.05 was considered statistically significant.

## Results

### Patient characteristics

A total of 1362 patients were included in this study. Table 1 summarizes the demographic and clinical information between the training and validation sets. In the entire follow-up cohort, the overall median survival time was 66-months (IQR: 25–112.75), with 66.5-months (IQR: 25–113) in the training set and 65.5-months (IQR: 25.75–110) in the validation set. During the follow-up period, a total of 677 patients (49.7%) died, comprising 472 patients (49.5%) in the training set and 205 patients (50.2%) in the validation set.

**Table 1:** Baseline characteristics of patients in the total dataset.

Variables	Total (n = 1362)	Training set (n = 954)	Validation set (n = 408)	*p*	Statistic
Status				0.841	0.04
Dead	677 (49.706)	472 (49.476)	205 (50.245)		
Alive	685 (50.294)	482 (50.524)	203 (49.755)		
Month	66 (25, 112.75)	66.5 (25, 113)	65.5 (25.75, 110)	0.805	196259.5
Age				0.093	4.751
<60	588 (43.172)	394 (41.3)	194 (47.549)		
60‒79	584 (42.878)	425 (44.549)	159 (38.971)		
≥80	190 (13.95)	135 (14.151)	55 (13.48)		
Sex				0.879	0.023
Male	707 (51.909)	497 (52.096)	210 (51.471)		
Female	655 (48.091)	457 (47.904)	198 (48.529)		
Race				0.118	4.279
White	1049 (77.019)	739 (77.463)	310 (75.98)		
Black	125 (9.178)	78 (8.176)	47 (11.52)		
Other	188 (13.803)	137 (14.361)	51 (12.5)		
Marital status				0.183	1.774
0	770 (56.535)	551 (57.757)	219 (53.676)		
1	592 (43.465)	403 (42.243)	189 (46.324)		
Laterality				0.812	0.056
0	666 (48.899)	469 (49.161)	197 (48.284)		
1	696 (51.101)	485 (50.839)	211 (51.716)		
Grade				0.853	1.347
I	132 (9.692)	87 (9.119)	45 (11.029)		
II	315 (23.128)	222 (23.27)	93 (22.794)		
III	309 (22.687)	216 (22.642)	93 (22.794)		
IV	145 (10.646)	104 (10.901)	41 (10.049)		
Unknown	461 (33.847)	325 (34.067)	136 (33.333)		
AJCC Stage				0.599	1.872
I	349 (25.624)	238 (24.948)	111 (27.206)		
II	275 (20.191)	188 (19.706)	87 (21.324)		
III	335 (24.596)	242 (25.367)	93 (22.794)		
IV	403 (29.589)	286 (29.979)	117 (28.676)		
T Stage				0.809	0.968
T1	387 (28.414)	264 (27.673)	123 (30.147)		
T2	370 (27.166)	261 (27.358)	109 (26.716)		
T3	435 (31.938)	310 (32.495)	125 (30.637)		
T4	170 (12.482)	119 (12.474)	51 (12.5)		
N Stage				0.334	Fisher
N0	945 (69.383)	660 (69.182)	285 (69.853)		
N1	157 (11.527)	103 (10.797)	54 (13.235)		
N2	252 (18.502)	184 (19.287)	68 (16.667)		
N3	8 (0.587)	7 (0.734)	1 (0.245)		
M Stage				0.841	0.04
M0	1273 (93.465)	893 (93.606)	380 (93.137)		
M1	89 (6.535)	61 (6.394)	28 (6.863)		
Surgery				0.252	1.31
No	138 (10.132)	103 (10.797)	35 (8.578)		
Yes	1224 (89.868)	851 (89.203)	373 (91.422)		
Radiation				0.923	0.009
No	495 (36.344)	348 (36.478)	147 (36.029)		
Yes	867 (63.656)	606 (63.522)	261 (63.971)		
Chemotherapy				0.025	5.008
No	1143 (83.921)	815 (85.43)	328 (80.392)		
Yes	219 (16.079)	139 (14.57)	80 (19.608)		
Tumor size				0.939	0.406
≤2	459 (33.7)	318 (33.333)	141 (34.559)		
2 < −4	562 (41.263)	398 (41.719)	164 (40.196)		
>4	288 (21.145)	200 (20.964)	88 (21.569)		
Unknown	53 (3.891)	38 (3.983)	15 (3.676)		
Histologic types				0.525	3.202
Adenoid cystic carcinoma	440 (32.305)	318 (33.333)	122 (29.902)		
Mucoepidermoid carcinoma	231 (16.96)	167 (17.505)	64 (15.686)		
Squamous cell carcinoma	174 (12.775)	118 (12.369)	56 (13.725)		
Adenocarcinoma	112 (8.223)	77 (8.071)	35 (8.578)		
Other	405 (29.736)	274 (28.721)	131 (32.108)		

### Model construction

**Fig. 2:**
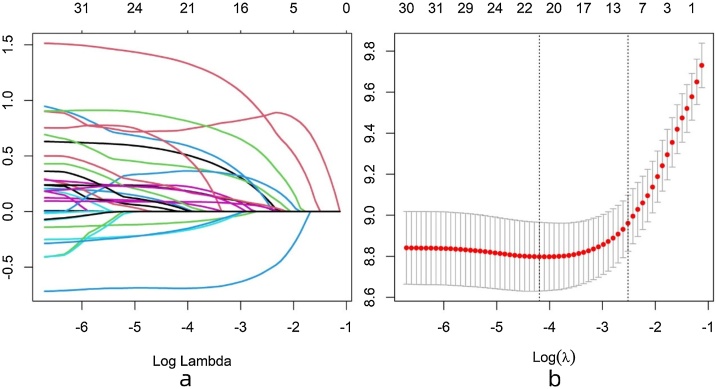
Selection of variables from 21 predictors by LASSO regression analysis. (a) Cross-validation plot, use 10-fold cross-validation to identify the best penalty coefficient λ in the LASSO model. (b) The coefficient profile: By obtain the best λ, nine variables with non-zero coefficients are selected.

LASSO regression analysis was performed, and significant variables were selected using lambda.1se. The selected significant variables included “Age”, “Grade”, “AJCC_Stage”, “T_Stage”, “N_Stage”, “M_Stage”, “Surgery”, “Chemotherapy”, and “Tumor_size”. The variable selection process is illustrated in Fig. 2. Multivariable Cox regression analysis demonstrated that “Age”, “Grade”, “AJCC_Stage”, “T_Stage”, “N_Stage”, “M_Stage”, “Surgery”, and “Tumor_size” were prognostic factors for SGC. The details of the multivariable Cox regression analysis are shown in Table 2.

**Table 2:** Baseline characteristics of patients in the total dataset.

Variables	HR	95% CI	p
**Age**			
<60	Reference		
60‒79	1.852	1.481‒2.316	<0.001
≥80	4.633	3.521‒6.096	<0.001
Grade			
I	Reference		
II	1.561	0.962‒2.534	0.072
III	2.368	1.484‒3.780	<0.001
IV	2.907	1.769‒4.778	<0.001
Unknown	1.452	0.914‒2.307	0.115
AJCC Stage			
I	Reference		
II	1.497	0.779‒2.877	0.226
III	1.434	0.797‒2.579	0.229
IV	2.897	1.526‒5.498	0.001
T Stage			
T1	Reference		
T2	0.486	0.237‒0.996	0.049
T3	0.818	0.438‒1.528	0.529
T4	0.505	0.254‒1.001	0.050
N Stage			
N0	Reference		
N1	1.497	1.085‒2.065	0.014
N2	1.329	0.940‒1.877	0.107
N3	2.639	0.987‒7.055	0.053
M Stage			
M0	Reference		
M1	2.506	1.768‒3.553	<0.001
Surgery			
No	Reference		
Yes	0.475	0.357‒0.632	<0.001
Chemotherapy			
No	Reference		
Yes	1.108	0.855‒1.437	0.439
Tumor size			
≤2	Reference		
2 < −4	1.675	1.049‒2.674	0.031
>4	1.798	1.143‒2.827	0.011
Unknown	1.732	0.998‒3.006	0.051

**Fig. 3:**
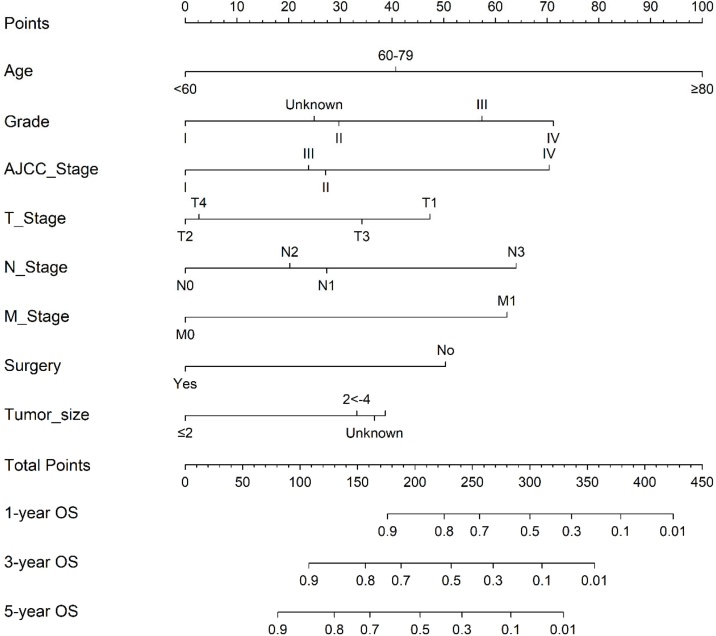
Nomogram of predicting 1-year, 3-year, and 5-year OS in SGC patients.

This study also incorporated clinically commonly used variables such as “T Stage”, “N Stage”, and “M Stage” into the Cox proportional risk model modeling as controls. Fig. 3 shows a column chart for predicting the 1-year, 3-year, and 5-year survival risk of SGC based on the Cox model. In addition, for the convenience of calculating individual probabilities, the scores based on column charts for all variables are shown in Table 3. We found that Age had the greatest impact on SGC, followed by Grade, AJCC Stage, and others.

**Table 3:** Score of each feature in nomogram.

Variables	Classifications	Scores
Age	<60	0
	60‒79	40.7
	≥80	100
Grade	I	0
	II	29.7
	III	57.4
	IV	71.2
	Unknown	24.9
AJCC Stage	I	0
	II	27.2
	III	23.9
	IV	70.4
T Stage	T1	47.3
	T2	0
	T3	34.2
	T4	2.7
N Stage	N0	0
	N1	27.4
	N2	20.2
	N3	64
M Stage	M0	0
	M1	62.2
Surgery	No	50.4
	Yes	0
Tumor size	≤2	0
	2 < −4	33.2
	>4	38.7
	Unknown	36.6

### Model evaluation

**Fig. 4:**
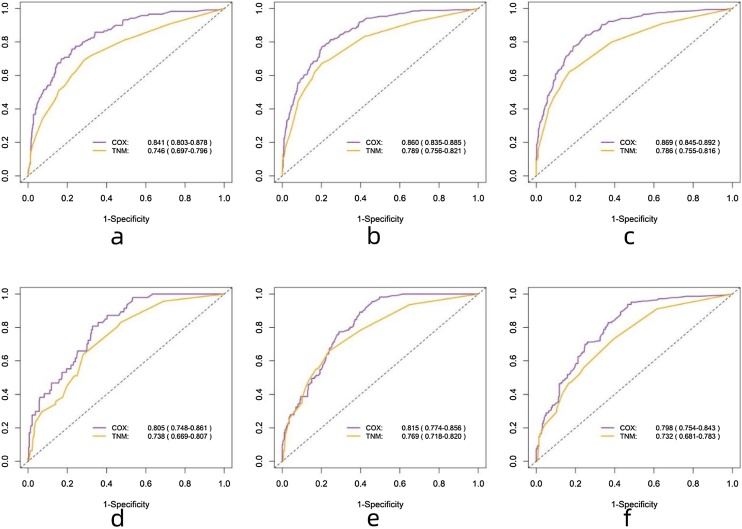
ROC curves of nomogram in the (a–c) 1-year, 3-year, and 5-year OS; training set and (d–f) 1-year, 3-year, and 5-year OS validation set.

**Fig. 5:**
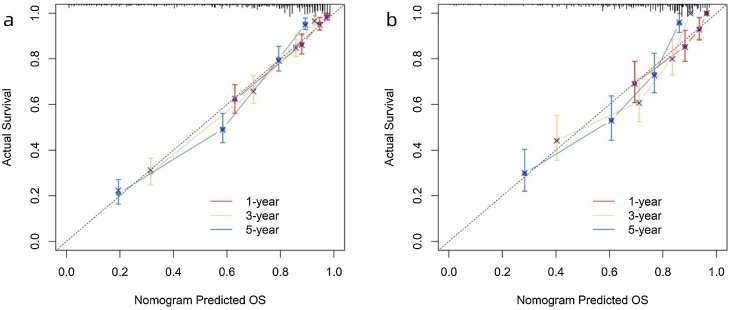
Calibration curves of nomogram in the (a) training set and (b) validation set.

In the training and validation sets, the ROC results are shown in Fig. 4, with the COX model's AUC significantly outperforming the TNM model. The c-index results are presented in Table 4, showing that the COX model's c-index is superior to the TNM model, indicating its excellent model discrimination ability. Additionally, the calibration plots are close to the 45° diagonal line, indicating that the columnar graph achieved good calibration (Fig. 5).

**Table 4:** C-index of nomogram in the (a) training set and (b) validation set.

	Training set	Validation set
COX model	0.802 (0.784‒0.821)	0.756 (0.725‒0.787)
TNM model	0.728 (0.705‒0.750)	0.693 (0.657‒0.728)

**Fig. 6:**
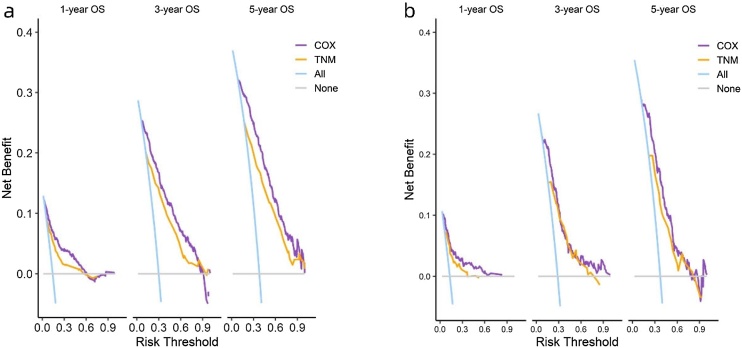
Comparison of DCA curves of nomogram in the (a) training set and (b) validation set.

The results of DCA are shown in Fig. 6. The Cox model demonstrates clinical net benefit over a time span of 1-year, 3-years, and 5-years, and it outperforms the TNM model.

### Patient risk stratification

**Fig. 7:**
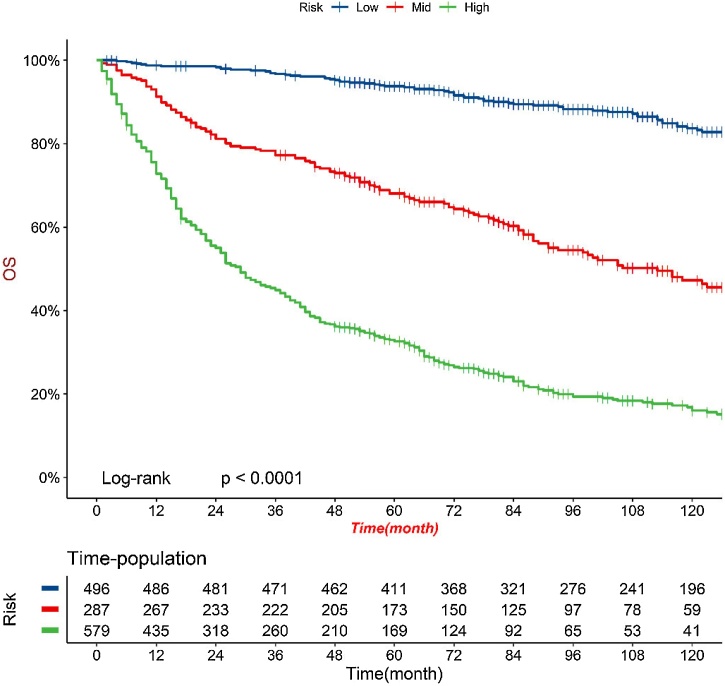
Cox risk stratification of patients in the total dataset.

Patient stratification is of great significance in guiding patient management. First, the training and validation sets were combined, and the columnar graph scores were calculated for all patients (Supplementary Material). The optimal cut-off value was calculated using the CatPredi package in R software to divide patients into high-risk group (risk score > 170), medium-risk group (121 ≤ risk score ≤ 170), and low-risk group (risk score < 121). Kaplan-Meier analysis and log-rank test results for the high-risk, medium-risk, and low-risk groups are shown in Fig. 7, with 5-year overall survival rates for low-risk, medium-risk, and high-risk groups being 82.9%, 60.3%, and 29.2%, respectively. There were statistically significant differences in overall survival among different risk groups, indicating the excellent stratification ability of our prediction model (*p* < 0.001).

## Discussion

We employed LASSO regression combined with multivariable Cox regression to identify significant predictive factors for patient mortality and developed a columnar graph to predict individual probabilities of death in submandibular gland tumor patients. LASSO regression possesses strong feature selection capabilities by sparsifying features, thus identifying crucial predictors that influence prognosis, making it widely applied in feature selection for high-dimensional data.^11^ The Cox proportional hazards model is used to analyze survival data that vary over time. Proper application of the Cox proportional hazards model relies on understanding the unique aspects of survival analyses, including censorship and time-to-event outcomes.^12^ The Cox proportional hazards model takes into account the time factor and interprets the relative impact of different features on prognosis through Hazard Ratios (HR).

We found that age, tumor grade, AJCC stage, TNM stage, surgery, and tumor size were significant predictive factors for mortality, which is consistent with previous studies.^13^, ^14^, ^15^, ^16^ In the study by Won et al., they investigated the survival of 99 SGC patients and reported that tumor grade and TNM stage were important prognostic factors for these patients.^13^ Liu et al.'s research revealed that pathological grade and extraglandular extension were independent prognostic factors for Disease-Free Survival (DFS), while pN status and extraglandular extension were independent prognostic factors impacting Overall Survival (OS).^14^ Bhattacharyya et al.'s study with 370 patients suggested that younger age, decreased tumor grade, and radiation therapy improved survival, while gender, tumor size, extraglandular extension, and nodal positivity did not statistically influence survival.^15^ This is similar to our research conclusion, although it contradicts the influence of tumor size on prognosis. We acknowledge that postoperative radiotherapy may be an important survival factor. However, we did not include this factor in the current study mainly because the data on postoperative radiotherapy in the SEER database is incomplete. Additionally, existing literature shows some controversy regarding its impact on the prognosis of submandibular gland malignancies. Postoperative radiotherapy currently has little prognostic significance in low-grade SGC, but it has a positive effect on improving high-grade (highly malignant) SGC. For low-grade SGC with positive pathological factors, it is recommended to only undergo radical resection and there is no need for further radiotherapy.^16^, ^17^, ^18^, ^19^ Şahin et al.'s retrospective analysis of 24 cases indicated that overall stage and Extraglandular Extension (EGE) were significant predictors of Locoregional Control (LRC), and positive nodal stage and positive surgical margin were significant predictors of OS.^20^ However, further investigation and more related research are still needed to confirm these prognostic factors.

This study is the first analysis of mortality predictive factors in SGC, and the construction of a nomogram based on LASSO regression and Cox proportional hazards model. Compared to the TNM model, the individual nomogram model showed better predictive performance in terms of ROC curves, AUC values, C-indexes (0.802 for the training set and 0.756 for the validation set), and calibration curves, and the DCA results also confirmed its superior clinical utility. The risk stratification results demonstrated that the established scoring graph successfully identified low, medium, and high-risk patients in the dataset. Xingxi Luo et al. constructed a competing risk nomogram of Parotid gland carcinoma based on 7962 cases from the SEER database, with C-indexes of 0.84 (95% CI: 0.81–0.86) and 0.84 (95% CI: 0.82–0.86).^9^ Li L et al. developed nomogram models for OS and DFS of major salivary gland squamous cell carcinoma based on 815 cases from the SEER database, and the C-index of the nomogram for OS was higher than that of the AJCC stage system (0.71 vs. 0.59) and SEER combined stage (0.71 vs. 0.55).^21^ Compared to the models by Xingxi Luo and Li L, our study also obtained a good C-index and included radiation and chemotherapy data for investigation.

The SEER database has a large sample size, high data quality, and covers a diverse population, making it more representative, and the variables included in the model are generally readily available in hospital diagnostic and treatment records. Therefore, this study has strong clinical utility. However, it still has certain limitations. The exclusion of 1064 patients due to incomplete data may introduce selection bias. The study population in the SEER database is from the United States, and further research is needed to determine if the findings can be generalized to the East Asian population. Hence, the next step will involve external validation using patient data from local medical institutions.

## Conclusion

We have utilized SGC data from the SEER database for the first time and identified predictive factors for mortality risk. We constructed a Cox proportional hazards nomogram incorporating these predictive factors to estimate individual probabilities of death, providing clinicians with an effective tool for decision-making and personalized treatment in the management of SGC. The application of this model holds promising prospects for the future.

## Informed consent

Informed consent was waived due to approved use of publically accessible database and retrospective nature of the study.

## Ethical approval

All personal identifying information for patients was anonymized by Surveillance, Epidemiology, and End Results, so ethical approval and informed consent were waived.

## Declaration of competing interest

The authors declare no conflicts of interest.
